# Epidemiological investigation and ultrasonic diagnosis of developmental dysplasia of the hip in Chinese infants: A large multi-center cohort study

**DOI:** 10.1097/MD.0000000000028320

**Published:** 2022-01-14

**Authors:** Na Xu, Bei Xia, Hongwei Tao, Ke Sun, Qinghua Liu, Wenjuan Chen, Dan Wang, Hong Gao, Yan Guo, Yan Liu, Jun Gao, Jianbo Teng, Tianzi Li, Qiancheng He, Zhixia Wu

**Affiliations:** a Department of Ultrasound, The Shenzhen Children's Hospital, Shenzhen, Guangdong, China; b Department of Orthopedics, The Shenzhen Children's Hospital, Shenzhen, Guangdong, China; c Department of Ultrasound, The Qilu Children's Hospital of Shandong University, Jinan, Shandong, China; d Department of Ultrasound, The Hunan Children's Hospital, Changsha, Hunan, China; e Department of Ultrasound, The Zhengzhou Children's Hospital, Zhengzhou, Henan, China; f Department of Ultrasound, The Kunming Children's Hospital, Kunming, Yunnan, China; g Department of Ultrasound, The Shanxi Children's Hospital, Taiyuan, Shanxi, China; h Department of Ultrasound, The Guizhou Women and Children's Hospital, Guiyang, Guizhou, China; i Department of Ultrasound, The Wuhan Women's and Children's Health Care Center (Wuhan Children's Hospital), Wuhan, Hubei, China; j Shandong Medical Imaging Research Institute, Jinan, Shandong, China.

**Keywords:** developmental dysplasia of the hip, multi-center cohort study, risk factors

## Abstract

Developmental dysplasia of the hip (DDH) is common among Chinese infants, but a lack of large-scale, multi-center epidemiological studies has made it difficult to characterize the risk factors associated with this disease.This multi-center cohort study included 19,833 Chinese infants aged 14 days to 6 months. A multi-center ultrasound protocol was used to diagnose hip abnormalities, and epidemiological data of the infants were collected through questionnaires. Categorical variables were expressed as percentages and compared using χ^2^ test. Multivariate analysis was performed through logistic regression.

Of 19,833 infants, 345 had DDH (1.7%). DDH incidence was higher in female infants (n = 279) than in male infants (n = 66) (χ^2^ = 95.89, *P* < .05), and there were more left hip cases (n = 149) than right hip cases (n = 79) (χ^2^ = 12.49, *P* < .05). DDH incidence was statistically different amongst different age groups in months (χ^2^ = 451.71, *P* < .05), and it gradually decreased with age (*P* < .05). The prevalence of a positive DDH family history, breech presentation, oligohydramnios, swaddling style, and other musculoskeletal deformities was higher in the positive group than in the negative group (all *P* < .05). No significant differences were found in terms of delivery by cesarean section, multiple births, or premature birth between both groups.

Family history, breech presentation, oligohydramnios, musculoskeletal deformities, and female sex are high-risk factors for DDH in Chinese infants. The incidence of DDH gradually decreases with age. The results of this study provide evidence for the epidemiology of infant DDH in China.

## 1 Introduction

Developmental dysplasia of the hip (DDH) is a common disease among infants that can be prevented during development. Because of the high prevalence of DDH, neonatal clinical screening is being carried out in some European regions.^[1,2]^ In North America, a combination of screening for high-risk patients and clinical physical examination has been adopted as a model for diagnosing DDH. China has a large population, with 15.23 million births in 2018 alone. The country has a very large land area, and the different living habits and swaddling styles in different regions may lead to differences in the incidence and pathological types of DDH.

A standardized DDH ultrasound diagnosis multi-center prospective study collaboration group (DDH-SUSC) comprising ultrasound departments of 8 children's medical centers conducted DDH research on Chinese infants, including those from the northern, southern, and Central Plains regions. This multi-center, multi-region DDH ultrasound research was conducted based on the expert consensus on the normal reference value of the hip joint and ultrasonography in Chinese infants.

According to the DDH-SUSC study protocol, all infants were examined by ultrasound and classified based on DDH positivity or negativity. Further, factors affecting DDH positivity or negativity were compared to improve the prevention and treatment of DDH.

## 2 Materials and methods

### 2.1 Study participants and patient recruitment

From August 2017 to August 2018, the DDH-SUSC collaboration group examined 19,833 infants between the ages of 14 days and 6 months who met the inclusion criteria. The inclusion criteria were a principal diagnosis of DDH (code 71) according to the International Classification of Diseases 10th Revision, Clinical Modification (DDH classification was performed using the DDH-SUSC protocol); age between 14 days and 6 months; healthy, hospitalized, or outpatient status and with an application for ultrasound examination of the bilateral hip joint and stability test; and no nervous system abnormality on physical examination. The exclusion criteria were pathological dislocation, paralytic dislocation, spastic dislocation of the hip joint, and teratoid dislocation.

This multi-center study was approved by the ethics committee of Shenzhen Children's Hospital (approval number 2016 [002]); this study was registered in the Chinese Clinical Trial Registry (registration number ChiCTR-ODC-16008748). Patients’ legal guardians or next-of-kin provided written, informed consent, and the study protocol complies with the Declaration of Helsinki.

### 2.2 Data collection

**Figure 1:**
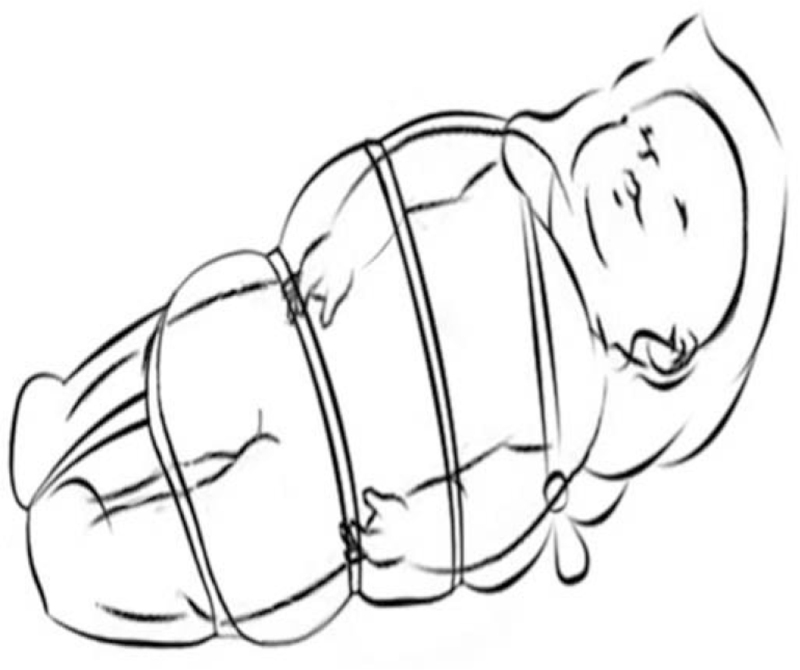
Swaddling mode (line diagram).

A questionnaire was designed to collect epidemiological data, such as infant's sex, length, weight, and nationality. Data on maternal pregnancy history (fetal position, oligohydramnios, parity, multiple births), birth history (preterm birth, gestational age, delivery mode), swaddling mode (Fig. 1), and other characteristics were collected.

### 2.3 Outcome assessment

#### 2.3.1 Experimental method

The DDH-SUSC collaboration group used GE Voluson E8, GE Logic E9 (General Electric Company, Boston, MA), Philips IU 22, Philips IU Elite, Philips CX50, Philips Epiq5 (Royal Philips, Amsterdam, Netherlands), Mindray DC-7 (Mindray Medical International, Shenzhen, China), ESaote Mylab (ESaote Company, Genoa, Italy), Toshiba Aplio500, Toshiba AlokaF75 (Toshiba Corporation, Tokyo, Japan), Hitachi Vision Preirus (Hitachi Company, Tokyo, Japan), and other ultrasonic instruments with a high-frequency (6-12 MHz) linear array probe for ultrasound experiments.

#### 2.3.2 Preexperiment preparation

To achieve a unified study amongst all collaborating units, the lead hospital held DDH training courses in 2013 and 2017 and invited Professors Graf and Harcke to teach in person and provide guidance during the standardized operation. Unified physical examination and ultrasound examination standards were adopted in each center.^[3]^

#### 2.3.3 Operation and measurement

Ultrasound imaging was used to obtain the coronal, transverse, and flexion posture transverse views and to perform the dynamic stability test of the hip joint.^[3]^ The α angle and the distance between the femoral head and the acetabulum were measured. The development of the acetabulum, the stability or instability of the hip joint, and the positional relationship between the femoral head and the acetabulum were evaluated.

#### 2.3.4 Diagnostic criteria

**Figure 2:**
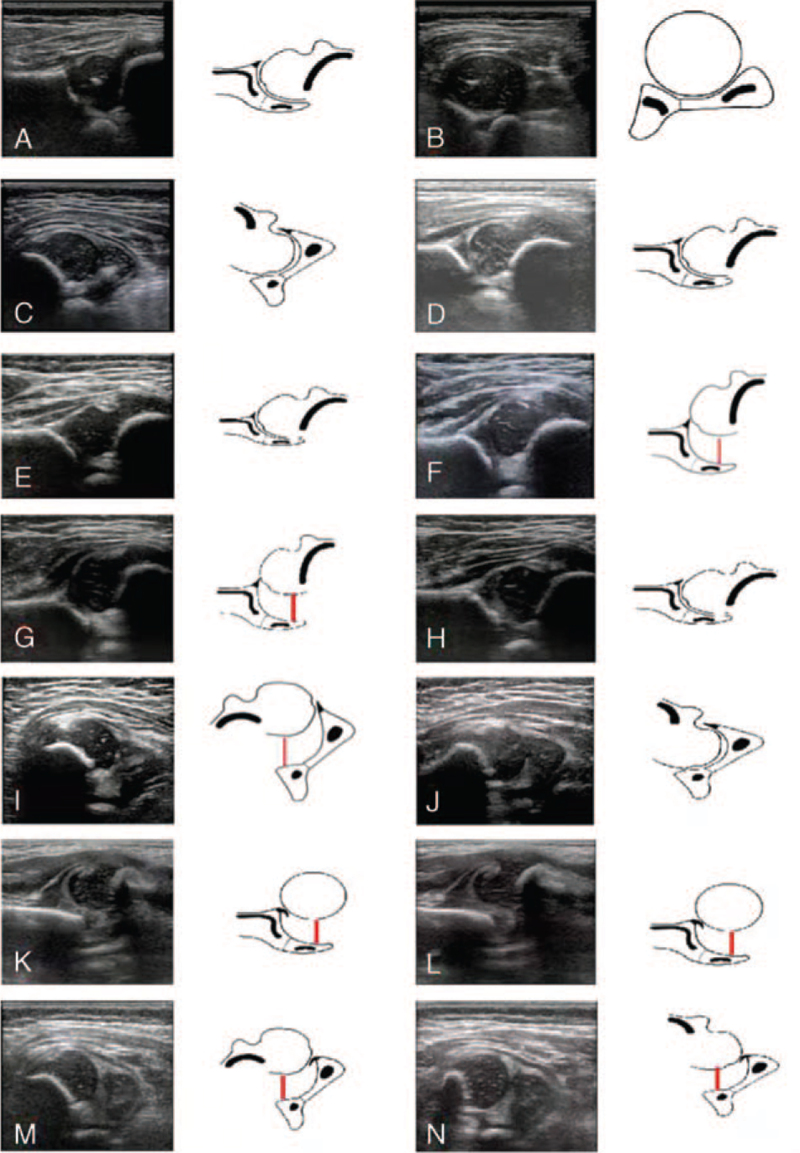
Ultrasonic image and schematic diagram of the hip joint. (A-C) Ultrasound examination of both hip joints in a 3-mo-old female infant shows no abnormality. (D) Physical examination shows non-symmetrical skin folds for both hip joints in a 1-mo-old female infant. Ultrasonography reveals that the right hip joint is immature (α angle <60°). (E) Physical examination shows non-symmetrical skin folds for both hip joints in a 4-mo-old male infant. Ultrasonography reveals that the left hip acetabulum is dysplastic (α angle <60°). (F) Physical examination shows asymmetrical skin folds for both hip joints and unequal thickness of bilateral thighs in a 2-mo-old female infant. Ultrasonography shows that the right hip is unstable. The pubo-femoral distance is increased (red line). (G-J) Physical examination shows asymmetrical skin folds for both hip joints, and the Ortolani test was positive in a 5-mo-old female infant. Ultrasonography shows a reducible dislocation of the left hip. (G, I) Increased pubo-femoral distance before the stability test (red line). (H, J) Decreased pubo-femoral distance after the stability test (red line). (K-N) Physical examination shows asymmetric skin folds for both hip joints, unequal leg lengths, and a positive Ortolani test in a 5-mo-old male infant. Ultrasonography shows irreducible dislocation of the left hip. (K, M) Increased pubo-femoral distance before the stability test (red line). (L, N) Decreased pubo-femoral distance after the stability test (red line).

The diagnostic criteria included ultrasonic classification of the hip joint at any stage: normal hip joint, immature hip joint (≤3 months), dysplasia of the hip joint (>3 months), unstable hip joint, reducible dislocation of the hip joint, and irreducible dislocation of the hip joint. Children with normal ultrasound results at any stage were assigned to the negative group, whereas those with an immature hip, hip dysplasia, hip instability, reducible hip dislocation, and irreducible hip dislocation were assigned to the positive group (Fig. 2, Table 1).

**Table 1:** DDH hip joint classification diagnosis table.

		Acetabular development	Femoral head and acetabulum position		
Diagnose	Age (mo)	ɑ angle	Femoral head coverage (neutral posture)	Femoral-pubo distance (flexion posture hip transverse section)	Pubo-femoral distance (transverse section)
Nomal	Any month	≥60°	≥50%	≤2.8 mm	≤3.5 mm
Immaturity	≤3	<60°	40%-49%	≤2.8 mm	≤3.5 mm
Dysplasia	>3	<60°	40%-49%	≤2.8 mm	≤3.5 mm
Instability	Any month	≥60°	Before axial stability test ≥50%	Before Barlow stability test ≤2.8 mm	
			After axial stability test <40%	After Barlow stability test >2.7 mm	
Dislocation	any month	≥60°or<60°	Before axial stability test <40%	>2.7 mm	>3.5 mm
Reducible			After axial stability test ≥50%		
Irreducible			After axial stability test < 40%		

### 2.4 Statistical analysis

The EpiData electronic database was used to collect, input, and manage the data of all participants; thereafter, all data were reviewed and analyzed. The incidence rate of DDH was compared between sexes, between the right and left sides of the hip, among degrees of DDH development (normal, immature, dysplasia, reducible dislocation, irreducible dislocation, and unstable), and among different age groups (<1 month and 2, 3, 4, 5, and 6 months). Differences in family history, breech delivery, oligohydramnios, swaddling, and other skeletal deformities between the positive and negative groups were compared. Categorical variables were expressed as percentages and compared using the χ^2^ test. Logistic regression was used in the multivariate analysis. All statistical analyses were performed using SPSS version 22.0 (IBM Corp., Armonk, NY). The results were considered statistically significant at *P* < .05.

## 3 Results

### 3.1 Comparison of the incidence rate of DDH between sexes and between the right and left sides of the hip joint

**Figure 3:**
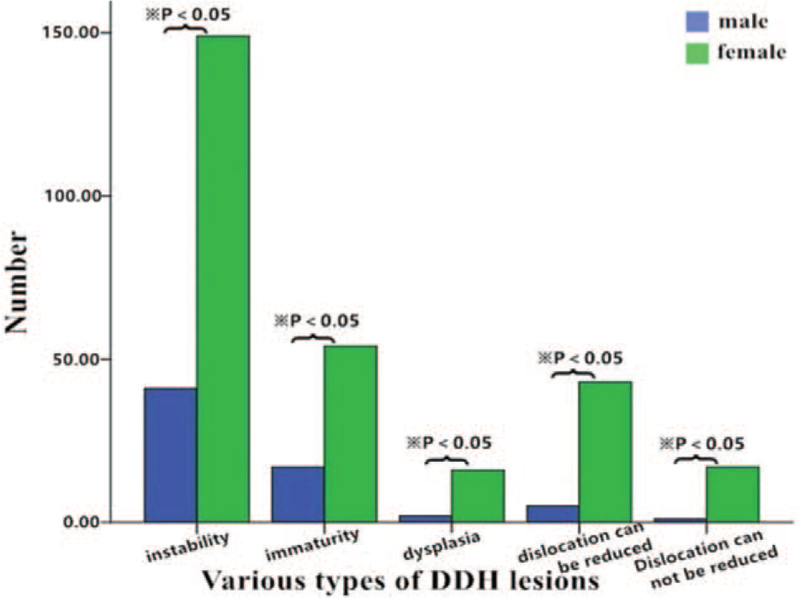
Distribution of different types of developmental dysplasia of the hip in male and female infants.

Among the 19,488 infants in the negative group, 10,602 were female and 8886 were male, and only 15,590 had complete epidemiological data. Of the 345 infants with DDH, 279 were female and 66 were male, and only 303 had complete epidemiological data. The DDH detection rate was higher in female participants (0.03; 95% confidence interval [CI], 0.02-0.03) than in male participants (0.01; 95% CI, 0.01-0.01), and this difference was statistically significant (χ^2^ = 95.89, *P* < .05) (Fig. 3, Table 2).

**Table 2:** Detection results of various types of DDH lesions by gender, left or right side.

	Gender			Side		
Classification	Male	Female	Total	Left	Right	Total
Instability	41 (21.6)	149 (78.4)	190 (100.0)	141 (52.6)	127 (47.4)	268 (100.0)
Immature	17 (23.9)	54 (76.1)	71 (100.0)	64 (59.8)	43 (40.2)	107 (100.0)
Dysplastic	2 (11.1)	16 (88.9)	18 (100.0)	13 (65.0)	7 (35.0)	20 (100.0)
Reducible dislocation	5 (10.4)	43 (89.6)	48 (100.0)	42 (80.8)	10 (19.2)	52 (100.0)
Irreducible dislocation	1 (5.6)	17 (94.4)	18 (100.0)	10 (52.6)	9 (47.4)	19 (100.0)
Total	66 (19.1)	279 (80.9)	345 (100.0)	270 (57.9)	196 (42.1)	466 (100.0)

Data are number and percentage (%).

**Figure 4:**
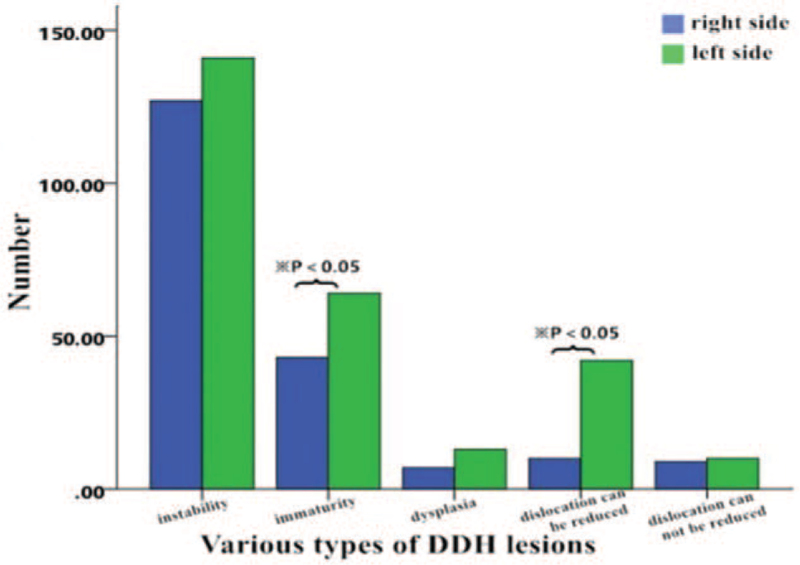
Distribution of different types of developmental dysplasia of the hip on the left and right hip joints.

A total of 148 cases of DDH were found in the left hip joint (43.0%), 74 in the right hip joint (21.5%), and 122 in both hip joints (35.5%). The DDH detection rate was significantly different between the left (0.01; 95% CI, 0.01-0.02) and right hip joints (0.01; 95% CI, 0.01-0.01) (χ^2^ = 12.49, *P* < .05) (Fig. 4).

### 3.2 Classification diagnosis results

A total of 466 diseased joints were detected in 39,666 hip joints, accounting for 1.2% (466/39,666) of the total. Among the 466 diseased joints, 107 were cases of hip joint immaturity, 20 were dysplasias, 52 were reducible dislocations, 19 were irreducible dislocations, and 268 were cases of joint instability. The incidence rate of diseased joints among different age groups was significantly different (χ^2^ = 451.71, *P* < .05). The incidence rate of all types of DDH lesions gradually decreased with age (<1 month:0.25, 95% CI, 0.20-0.31; <2 months:0.07, 95% CI, 0.06-0.08; <3 months:0.03, 95% CI, 0.02-0.04; <4 months:0.01, 95% CI, 0.01-0.01; <5 months:0.01, 95% CI, 0.01-0.01; <6 months: 0.01, 95% CI, 0.01-0.01) (*P* < .05) (Table 3).

**Table 3:** The positive incidence rate of hip ultrasound in infants of different months of age.

			OR (95% CI)				
Age (mo)	Case/N	Prevalence (%)	Immaturity	Dysplasia	Reducible dislocation	Irreducible dislocation	Instablity
<1	54/217	33.0	10.60 (7.17-15.40)	0	0.92 (0.25-3.30)	0.92 (0.25-3.30)	12.44 (8.69-17.49)
<2	109/1621	7.0	2.47 (1.82-3.35)	0	0.74 (0.42-1.29)	0.06 (0.01-0.35)	3.45 (2.67-4.45)
<3	45/1461	3.0	0.55 (0.28-1.08)	0	0.48 (0.23-0.99)	0.14 (0.04-0.50)	1.92 (1.33-2.76)
<4	70/8489	1.0	0	0.12 (0.07-0.22)	0.18 (0.11-0.30)	0.06 (0.03-0.14)	0.47 (0.35-0.64)
<5	46/4749	1.0	0	0.08 (0.03-0.21)	0.21 (0.11-0.39)	0.11 (0.05-0.25)	0.57 (0.39-0.83)
<6	21/3296	1.0	0	0.12 (0.05-0.31)	0.06 (0.02-0.22)	0.09 (0.03-0.27)	0.36 (0.21-0.63)

### 3.3 Risk factors

**Figure 5:**
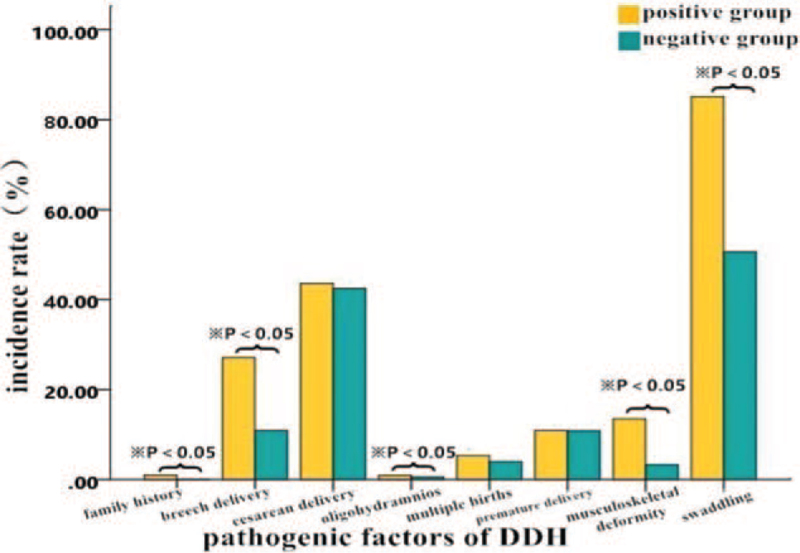
Comparison of developmental dysplasia of the hip prevalence rates and risk factors between the positive and negative groups.

Information on the distribution characteristics of risk factors in the positive and negative groups is shown in Table 4. The incidence rates of a DDH family history (odds ratio [OR], 17.94; 95% CI, 1.44-222.87), breech presentation (OR, 3.68; 95% CI, 1.34-–10.10), oligohydramnios (OR, 200.52; 95% CI, 40.14-1001.58), swaddling (OR, 43.07; 95% CI, 18.05-102.77), and musculoskeletal deformity (OR, 6.31; 95% CI, 1.99-20.00) were higher in the positive group than in the negative group (all *P* < .05). However, there were no significant differences between the 2 groups in terms of cesarean delivery, multiple births, and premature delivery (all *P* > .05) (Table 4, Fig. 5).

**Table 4:** Logistic multivariate regression analysis of pathogenic factors of DDH.

Risk factors	Positive group (%)	Negative group (%)	β value	Wold value	OR value	*P* value	95% CI
Family history	3 (1.0)	15 (0.1)	2.89	5.04	17.94	.025	1.44, 222.87
Breech delivery	82 (27.1)	1699 (10.9)	1.30	6.39	3.68	.011	1.34, 10.10
Cesarean delivery	132 (43.6)	6625 (42.5)	0.93	3.21	2.54	.073	0.92, 7.06
Oligohydramnios	31 (0.9)	93 (0.6)	5.30	41.73	200.52	<.001	40.14, 1001.58
Multiple births	16 (5.3)	625 (4.0)	0.51	0.58	1.66	.445	0.45, 6.07
Premature delivery	33 (10.9)	1683 (10.8)	0.53	1.51	1.70	.219	0.73, 3.96
Musculoskeletal deformity	41 (13.5)	514 (3.3)	1.84	9.79	6.31	.002	1.99, 20.00
Swaddling	258 (85.1)	7888 (50.6)	3.76	71.93	43.07	<.001	18.05, 102.77

*P* <.05, the difference was statistically significant.

## 4 Discussion

DDH was previously called congenital dislocation of the hip and dysplasia of the hip. However, the Pediatric Orthopaedic Society of North America proposed that the condition be renamed developmental dysplasia of the hip in 1992. The name change also clearly shows that the disease can occur naturally, that is, the hip joints can be normal at birth, with DDH developing gradually as the infant grows. Hip joints can also be abnormal at birth; some cases naturally develop into normal hip joints, but some require intervention and treatment if there is no improvement after some time. Ultrasound has become the first-choice examination for DDH and is an effective tool for early DDH diagnosis.

Different methods for infant hip ultrasound imaging are used in different regions of China, including the Graf method, Harcke method, and others. Therefore, recognising DDH varies greatly in different regions and remains controversial. This multi-center, multi-region DDH hip ultrasound research, which was conducted to highlight the role of ultrasound in DDH diagnosis and improve the prevention and treatment of DDH, was based on expert consensus on the normal reference values for Chinese infant hip ultrasound imaging and diagnosis methodology.

### 4.1 DDH risk factors

The incidence rates of DDH reported worldwide are significantly different, ranging from 1.5% to 20.0%.^[4]^ The incidence per 1000 live births ranges from 0.06 in Africans to 76.1 in Native Americans.^[5]^ The incidence of DDH is highest in Native Americans, whereas it is significantly lower in the African population. The sample data from this study indicate a DDH incidence rate of 1.7%, which is relatively low. Although a high DDH incidence rate has been reported in a previous study, only 0.5% of hip abnormalities require treatment, and these are classified as real DDHs.^[6]^ Hence, the DDH incidence rate might have been overestimated in the past. However, this rate may also be influenced by race and genes, explaining the low DDH incidence rate in China.

Previous studies have suggested that factors such as breech presentation, female sex, first child, oligohydramnios, and a positive DDH family history may increase DDH risk.^[7–12]^ Other factors include plantar adduction, torticollis, and swaddling style. The most important of these risk factors is breech delivery, followed by female sex or a positive DDH family history.^[7,8,10–13]^ In this study, the analysis of the DDH in Chinese infants revealed that oligohydramnios, swaddling style, other deformities, and breech delivery were the main risk factors. However, the most common risk factor was oligohydramnios, followed by swaddling style, a positive DDH family history, musculoskeletal deformity, breech delivery, delivery by cesarean section, premature delivery, and multiple births. The amniotic fluid protects the fetus from the external environment and allows the fetus to have a certain activity space. When the fetal size increases, the amniotic fluid volume gradually decreases, and the fetus is subjected to mechanical pressure from the uterus and the abdominal wall, resulting in hip dislocation. Oligohydramnios and multiple births, especially when complicated with other postural malformations (such as torticollis, metatarsal adduction, and talipes equinovarus), suggest that DDH is related to intrauterine mechanical extrusion. In cases of breech presentation, the fetal hip drops during delivery, the fetal hip joint touches the rear of the maternal pubic symphysis, and the hip continues to lower under labor pressure, while the lower limbs straighten and stick in front of the chest and abdomen. In the case of resistance of the birth canal, the limbs tend to separate from the hip joint, and the joint capsule elongates, resulting in dislocation. In this study, 1.0% of infants with DDH had a positive family history of the disorder. Meanwhile, infants who did not have DDH but had a positive family history of DDH only accounted for 0.1%. This difference suggests that, for Chinese infants, a family history of DDH is one of the risk factors. Further, a strong correlation exists between environmental factors, such as swaddling style after birth, and hip development. In the traditional swaddling method, the lower limbs are not in the abduction state; instead, they are straightened and wrapped too tightly, affecting hip joint development.^[7]^ This poor swaddling style was once common in the cold northeast regions of China and Japan.^[14]^ These results show that risk factors for DDH in different regions and among different races are dependent on genes and lifestyles.

In this study, DDH morbidity was higher in female infants than in male infants, consistent with previous research results.^[14–18]^ This trend may be related to the relaxation of the hip joint capsule and surrounding ligaments in female infants due to endocrine factors.^[19]^ In addition, DDH incidence was higher in the left hip joint than in the right hip joint.^[20]^ This may be because most fetuses are in the left occiput anterior position at the time of birth. In this position, the left hip of the fetus is adjacent to the mother's sacrum, predisposing it to take an adduction position.^[21]^

### 4.2 Unstable hip joint

The present study results show that the DDH prevalence was highest in infants younger than 1 month, reaching 33.1%. The number of positive DDH cases was highest in infants aged 1 to 2 months, with a total of 109 cases, which were mainly cases of hip instability. These results show that late diagnosis reduces the possibility of treatment; hence, early and reasonable diagnosis is necessary.

Whether an unstable hip joint should be classified as DDH or an early change in DDH has always been controversial. Most unstable hip joints can gradually become normal as the infant grows; however, some instabilities remain. For example, with hip joint instability, friction between the acetabulum and the femoral head results in the deformation of both, causing dislocation. In this study, there were 170 cases of unstable hip joints. We will continue to follow-up these infants for a year to ascertain the outcomes of these 170 children.

### 4.3 Limitations

This study has some limitations. All infants examined were those who visited the hospital for physical examination; the parents of infants in this group were often highly educated, and this might have caused a bias in selection.

## 5 Conclusions

DDH is a common deformity of the hip joint in children. The results of this multi-center cohort study showed that the incidence rate of DDH was 1.7%, which gradually decreased as the infants develop between 14 days and 6 months. Infants with oligohydramnios, breech delivery, other skeletal deformities, and a positive DDH family history as well as female infants need to be examined early for prompt diagnosis and treatment.

## Acknowledgments

The authors thank Dr. Yang Zhao and Prof. Dongsheng Hu of Shenzhen University for medical statistics and methodological design.

## Author contributions

**Conceptualization:** Na Xu, Bei Xia.

**Data curation:** Na Xu, Bei Xia.

**Formal analysis:** Na Xu, Bei Xia.

**Investigation:** Na Xu, Bei Xia, Hongwei Tao, Ke Sun, Qinghua Liu, Wenjuan Chen, Dan Wang, Hong Gao, Yan Guo, Yan Liu, Jun Gao, Jianbo Teng, Tianzi Li, Qiancheng He, Zhixia Wu.

**Methodology:** Na Xu, Bei Xia, Hongwei Tao, Ke Sun, Qinghua Liu, Wenjuan Chen, Dan Wang, Hong Gao, Yan Guo, Yan Liu, Jun Gao, Jianbo Teng, Tianzi Li, Qiancheng He, Zhixia Wu.

**Project administration:** Na Xu, Bei Xia.

**Resources:** Na Xu, Bei Xia.

**Supervision:** Bei Xia.

**Writing – original draft:** Na Xu, Bei Xia.

**Writing – review & editing:** Na Xu, Bei Xia, Hongwei Tao, Ke Sun, Qinghua Liu, Wenjuan Chen, Dan Wang, Hong Gao, Yan Guo, Yan Liu, Jun Gao, Jianbo Teng, Tianzi Li, Qiancheng He, Zhixia Wu.
